# Enhanced Optical Response of SnS/SnS_2_ Layered Heterostructure

**DOI:** 10.3390/s23104976

**Published:** 2023-05-22

**Authors:** Der-Yuh Lin, Hung-Pin Hsu, Kuang-Hsin Liu, Po-Hung Wu, Yu-Tai Shih, Ya-Fen Wu, Yi-Ping Wang, Chia-Feng Lin

**Affiliations:** 1 Department of Electronic Engineering, National Changhua University of Education, No. 2, Shi-Da Rd., Changhua 500, Taiwan; 2 Department of Electronic Engineering, Ming Chi University of Technology, No. 84, Gongzhuan Rd., Taishan Dist., New Taipei City 243, Taiwanyp.wang@mail.mcut.edu.tw (Y.-P.W.); 3 Department of Electrical Engineering, National Dong Hwa University, No. 1, Sec. 2, Da Hsueh Rd., Shoufeng, Hualien 974, Taiwan; 4 Department of Physics, National Changhua University of Education, No. 1, Jin-De Rd., Changhua 500, Taiwan; ytshih@cc.ncue.edu.tw; 5 Department of Materials Science and Engineering, National Chung Hsing University, No. 145, Xingda Rd., South Dist., Taichung 402, Taiwan; cflin@dragon.nchu.edu.tw

**Keywords:** heterostructure, photoresponsivity, chemical vapor deposition

## Abstract

The SnS/SnS_2_ heterostructure was fabricated by the chemical vapor deposition method. The crystal structure properties of SnS_2_ and SnS were characterized by X-ray diffraction (XRD) pattern, Raman spectroscopy, and field emission scanning electron microscopy (FESEM). The frequency dependence photoconductivity explores its carrier kinetic decay process. The SnS/SnS_2_ heterostructure shows that the ratio of short time constant decay process reaches 0.729 with a time constant of 4.3 × 10^−4^ s. The power-dependent photoresponsivity investigates the mechanism of electron–hole pair recombination. The results indicate that the photoresponsivity of the SnS/SnS_2_ heterostructure has been increased to 7.31 × 10^−3^ A/W, representing a significant enhancement of approximately 7 times that of the individual films. The results show the optical response speed has been improved by using the SnS/SnS_2_ heterostructure. These results indicate an application potential of the layered SnS/SnS_2_ heterostructure for photodetection. This research provides valuable insights into the preparation of the heterostructure composed of SnS and SnS_2_, and presents an approach for designing high-performance photodetection devices.

## 1. Introduction

Two-dimensional (2D) van der Waals materials have attracted a lot of attention due to their distinct optical and electrical properties [1,2,3,4,5]. Graphene has been the most investigated layered material in recent years due to its specific physical [6,7]. However, graphene is a zero band gap material, which limits its applications in electronic devices. Hence, 2D materials with semiconducting properties are in demand for next generation electronic devices [8]. The 2D layered materials with semiconductor behaviors can realize the thin and flexible requirements for the electronic devices’ applications. 2D semiconducting materials such as transition metal dichalcogenides (TMDs) [9], black phosphorus (BP) [10,11], NbOI_2_ [12], tellurene [13], SiAs [14], CuInP_2_S_6_ [15], and TaNi_2_Te_3_ [16] have been discovered as promising candidates for novel electronic devices in the future. Among these 2D layered materials, TMDs are the most studied materials due to their specific dimensional geometrics and distinct physical and optical properties [17,18]. To date, electronic devices fabricated by semiconducting 2D layered TMDs materials with layers such as p-n diode [19], field effect transistor [20], gigahertz frequencies FET [21], Fin shaped FET [22,23], and phototransistors [24] has been achieved. A 1-bit microprocessor with logical operations made by 2D TMDs have also been implemented [25].

SnS_2_ is a member of the 2D semiconductor family, and has a hexagonal CdI_2_ type crystal structure [26]. In recent studies, SnS_2_ has shown an n-type nature and has been shown to be a good candidate for many applications with good performance, such as photodetectors [27,28,29], sensors [30,31], and field-effect transistors [32,33]. However, for the devices’ applications, the block potential between n- and p-region heterostructures is an important issue [34], and hence studies on p-type dopants in SnS_2_ [35] or p-type SnO thin layers on n-type SnS_2_ nanosheets have also been reported [36]. It has been observed that devices made from layered materials using the exfoliation method tend to be smaller in size. The fabrication of large-sized optoelectronic devices is an important issue. SnS_2_ and SnS have a high potential use in optoelectronic and photoconductive devices. There are a number of methods for the growth of SnS_2_ and SnS films, such as dip coating [37], thermal evaporation [38], and chemical vapor deposition (CVD) [39]. Each method has its advantages and disadvantages; for example, the dip coating and spray pyrolysis methods provide low-cost thin semiconductor films. However, the film’s quality and uniformity may not be good [37]. Films grown by thermal evaporation may need post-growth annealing [38]. CVD is a widely used technology for the growth of thin films in which the amount of gas-phase precursors can be controlled by carrier gas, and thin films are deposited on a heated substrate controlled at a stable temperature. CVD offers an advantage by relying on chemical reactions that enable researchers to obtain high-quality and pure phase semiconductor films. Furthermore, CVD does not require high-vacuum environments, making it a popular technology for mass production [39]. Mechanical exfoliation is known as a useful technique to fabricate a monolayer photodetector or heterojunction devices with good performance in nano-dimensions, but it has complications for mass production or applications in solar energy with a large area. The large-area SnS/SnS_2_ heterostructure can be produced by CVD methods and paves the way for developing a solar panel on glass substrates which can be used for semi-transparent windows and also can provide power sources in buildings for the purpose of saving energy.

In this study, we adopt SnS as a p-type material and fabricate the SnS/SnS_2_ pn heterostructure by chemical vapor deposition methods. Here, we have successfully fabricated a centimeter-sized SnS/SnS_2_ pn heterostructure. The potential applications of SnS/SnS_2_ pn heterostructures for photodetection are explored. The SnS/SnS_2_ heterostructure was characterized by X-ray diffraction (XRD) and field emission scanning electron microscopy (FESEM), and the phase properties were achieved by Raman spectroscopy. Tests of their optical responses, such as absorption and photoresponsivity investigations, were also performed. The results of SnS/SnS_2_ heterostructure characterizations were determined and possible mechanisms were also discussed. The study proposes a method to enhance photodetection through the utilization of a SnS/SnS_2_ heterostructure.

## 2. Materials and Methods

The growth of SnS_2_ (CAS:1315-01-1), SnS (CAS:1314-95-0) thin films and the SnS/SnS_2_ heterostructure was carried out on glass substrates by the CVD method. The band gap energies of SnS_2_ and SnS cover the light spectra from the near-infrared to visible light range. At the same time, tin and sulfur are non-toxic and earth-abundant elements. Considering the impact on the environment and manufacturing cost, SnS_2_ and SnS thin films become potential candidates for realizing cheap solar cells. Glass is an optical transparency substrate with strong mechanical strength. For the same reason, glass is a suitable substrate not only for new-generation solar cells, but also for photodetectors. Thus, in this study we chose microscope glass (Matsunami Glass S1111 White Slide Glass) as a substrate. Glass substrates are cleaned by acetone and methanol, then put into an ultrasonic bath for 30 min to remove any contaminants on the glass. After that, we use a nitrogen gun to blow away any dust or residuals on the substrates.

A three-zone horizontal furnace is equipped with three temperature controllers to control the zone temperatures separately. The source materials including tin (II), chloride (SnCl_2_, 1.5 g) (CAS:7772-99-8), and elemental sulfur (CAS:7704-34-9) powder (S, 3.5 g) were placed in crucibles arranged in the center of zone one. Two electric heaters and temperature controllers were used to control the crucibles’ temperatures at 280 °C and 180 °C for SnCl_2_ and elemental S, respectively, to provide tin atoms and sulfur atoms. The glass substrates were arranged in zone three, about 30 cm away from the source materials. The zone one heater was set at 100 °C, and the zone two and zone three were set at 350 °C and 300 °C for the growth of SnS_2_ thin films, while for the growth of SnS thin film the zone temperatures were set at 150 °C and 100 °C, respectively. The carrier gas Ar (CAS:7440-37-1) flow rate was controlled by a mass flow controller and set at 70 sccm. When the zone temperatures reached stable values, the source materials were supplied constantly by heating the crucibles for 20 min. After film growth, the furnace was naturally cooled to room temperature under an Ar flow. For the growth of the SnS/SnS_2_ heterostructure, the SnS_2_ thin film was prepared in advance and then covered by a shadow mask for the growth of SnS thin films. Thus, the SnS_2_ and SnS films were stacked vertically. All the samples were grown to the size of ~10 × 10 mm^2^ in this study.

The XRD patterns were investigated by using a Rigaku D/max-2200 PC X-ray diffractometer. The morphology and crystal structure were investigated by a Hitachi S-4800 field emission scanning electron microscope (FESEM). The elemental compositions of SnS_2_ and SnS films were checked by energy-dispersive X-ray spectroscopy (EDS). The Raman spectroscopies were carried out on a Nanofinder 30 (Tokyo Instruments, Tokyo, Japan) three-dimensional laser Raman spectrometer equipped with a 532 nm laser. The laser power was operated at ~1 mW to avoid heating effects. For the measurements of the absorption spectra of the SnS_2_ and SnS thin film crystals and to determine their bandgaps, a 1/4 m monochromator (MKS, Irvine, CA, USA) was equipped with a Si photodetector with a sensing range of 1.1~3.1 eV. A 130 W halogen lamp was used to produce the light with a wide photon energy range and the monochromatic light was selected by the monochromator to illuminate the measured sample. The Si photodetector was used to receive the transmitted light.

**Figure 1:**
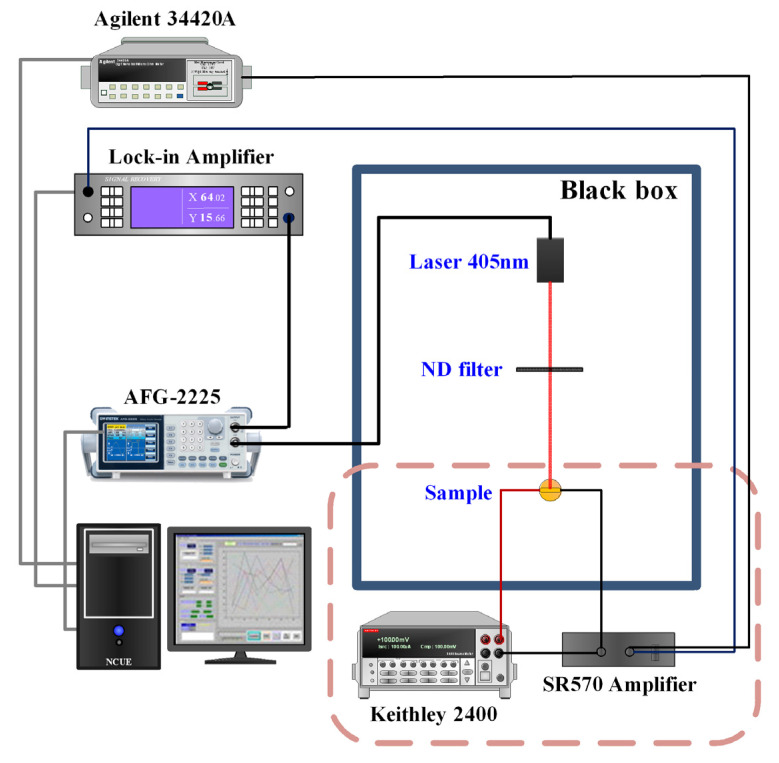
The schematic PC measurement setup.

Photoconductivity (PC) measurements can be taken at different illumination frequencies, illumination intensities, or bias voltages to examine the response-time constants and responsivity of the photosensitive materials. The system consisted of a diode laser (405 nm) controlled by a function generator (GW Instek AFG-2225) to provide an on/off light at different frequencies, and a lock-in amplifier (AMETEK Signal Recovery 7265 DSP Lock-in Amplifier) which was used to record the photo-induced current. A dc bias voltage was provided by a sourcemeter (Keithley 2400) and the photo-induced current was amplified by a low-noise current preamplifier (Stanford Research Systems SR 570) before feeding into the lock-in amplifier. A continuously variable neutral density filter wheel was employed to tune the illumination intensity of the laser diode for the PC measurements taken at different illumination intensities. A schematic optical measurement setup for the measurement of the photoconductivity at different bias voltages, illuminating frequencies or intensities, is shown in Figure 1.

## 3. Results and Discussion

**Figure 2:**
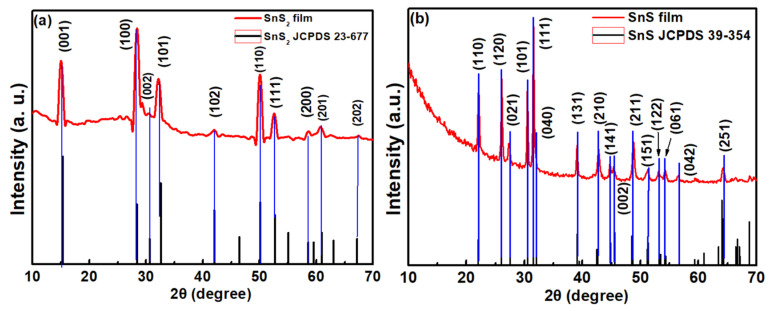
XRD patterns of (**a**) SnS2 and (**b**) SnS films.

Figure 2 depicts the XRD patterns of (a) SnS_2_ and (b) SnS films grown on glass substrates by chemical vapor deposition. The XRD patterns of SnS_2_ and SnS films reveal diffraction peak information deduced from different orientations. The various reflection peaks from the SnS_2_ film, such as (001), (100), and (101) planes, are identified in Figure 2a. In Figure 2b, the reflection peaks from various orientations from SnS are also indexed. The features were also confirmed with the JCPDS database (JCPDS 23-0677 and 39-0354). Table 1 lists the deduced lattice constants of SnS_2_ and SnS from XRD. The deduced lattice constants of SnS_2_ and SnS are in a reasonable agreement with the reported literature [40,41]. The growth of SnS_2_ thin films have a preferred orientation in the (001) plane, because the {001} plane of SnS_2_ has the lowest surface energy (g_100_ = 0.034 eV/Å^2^ and g_001_ = 0.0065 eV/Å^2^) [42]. In Figure 2a, the XRD pattern of SnS_2_ thin film shows that the intensities from (001), (100), and (110) are obviously higher than other planes. After forming the SnS_2_ nucleus, the new species favorably develop themselves along the [001], [010], and [110] directions due to the anisotropic nature of atomic bounding in SnS_2_ [43]. The small crystal may grow laterally before merging because of the selective reactions likely to happen at the crystal edges. The coalescence of different domains generates grain boundaries composed of dislocations. Subsequently, the growth changes into 3D mode, and develops vertically at the grain boundaries due to their higher reactivity than in the basal planes. Finally, the vertically oriented SnS_2_ nanosheet arrays with very high shape similarity are built which can be observed in the SEM image of the SnS_2_ thin film. SnS crystal has an orthorhombic structure. It consists of two layers stacked perpendicular to the c-axis, where ‘Sn’ and ‘S’ atoms are tightly bound in each layer, while the bonding between the layers is of a weak van der Waals type. The orthorhombic unit cell is similar to a nanobox, with three different edge lengths a, b, and c, which are at an angle of 90° to each other. The growth mechanism of SnS thin film could be simply understood as a block-building task. The SnCl_2_ and S atoms are constantly supplied from different quartz pipes. In the reaction zone, they are mixed and chemically transformed into an SnS compound to form the SnS nucleus with a nanobox shape. The carrier gas blows them toward the deposition zone, where they can be adsorbed on the glass substrate. These orthorhombic nanoboxes are stacked in a preferred orientation along (010) plane. The multiple peaks in the XRD pattern of SnS thin film shown in Figure 2b indicates that the film is polycrystalline and can be indexed based on an orthorhombic SnS (JCPDS 39-0354) cell. It is noteworthy to mention that XRD peaks show the strong intensity of the (111) plane because the lattice planes with lower surface energy tend to dominate the growth mechanism. Here, the more closely packed (111) crystallographic plane is preferred over the others [44].

**Figure 3:**
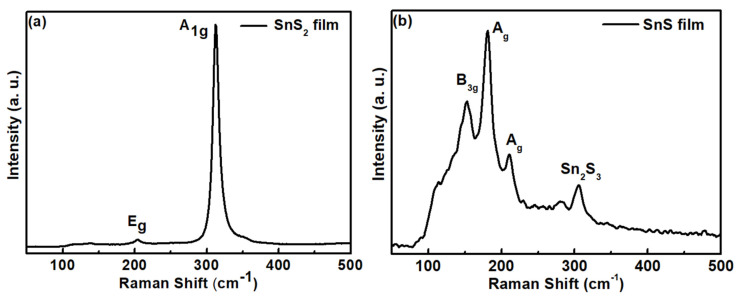
Raman spectra of (**a**) SnS_2_ and (**b**) SnS films.

Raman spectra of (a) SnS_2_ and (b) SnS films in the range of 50–500 cm^−1^ are shown in Figure 3. For SnS_2_ film, there are two peaks, which were identified as E_g_ and A_1g_ active modes [45]. For SnS film, the Raman peaks at 181 cm^−1^ and 210 cm^−1^ are associated with the A_g_ modes, while the peak at 152 cm^−1^ is assigned to B_3g_ mode [46]. An additional mode, at 306 cm^−1^, might come from the Sn_2_S_3_ phase [47].

**Figure 4:**
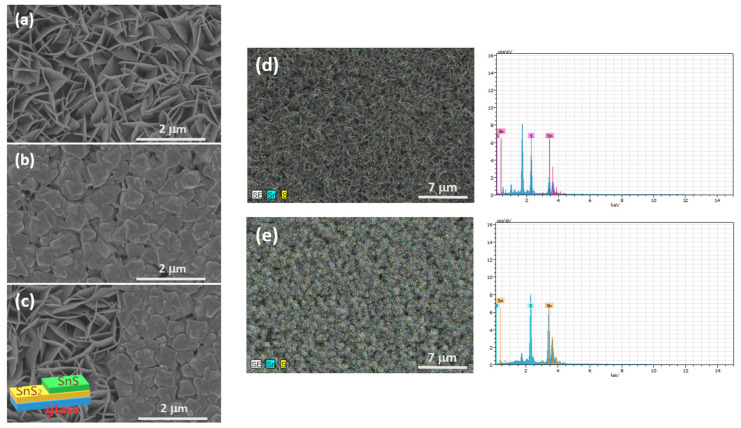
SEM image of (**a**) SnS_2_, (**b**) SnS films, and (**c**) SnS/SnS_2_ heterostructure. EDS measurements results of (**d**) SnS_2_ and (**e**) SnS films.

Figure 4a–c shows the FESEM images of (a) SnS_2_ films, (b) SnS films, and (c) the SnS/SnS_2_ heterostructure grown on the glass substrate by chemical vapor deposition. As shown in Figure 4a, the SnS_2_ film displays a leaf-like shape morphology. The morphology exhibits a hexagonal shape which indicates the typical layered structure of SnS_2_ film. Figure 4b shows the FESEM image of SnS film, where the morphology of cluster grains can be observed. The composition of SnS_2_ and SnS films were also confirmed by EDS measurement. Figure 4d,e shows the EDS measurement results of SnS_2_ and SnS films. In this study, the alloy elemental ratio determined by EDS of SnS_2_ film is Sn:S = 35%:65% and for SnS film it is Sn:S = 47%:53%. From the obtained results, it can be shown that the composition of the grown film is in a reasonable agreement with the nominal composition. In Figure 4c, the morphology image at the interface of the SnS/SnS_2_ heterostructure is displayed (the schematic diagram of SnS/SnS_2_ heterostructure is shown in the inset). From the FESEM images combined with XRD and Raman characterization, we can confirm that the SnS/SnS_2_ heterostructure was successfully fabricated.

**Figure 5:**
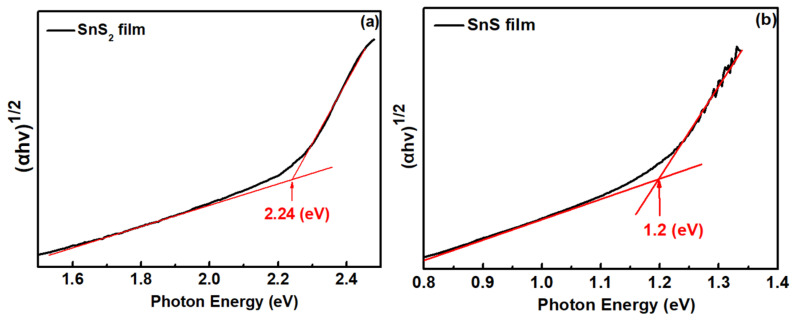
Absorption spectra of (**a**) SnS_2_ and (**b**) SnS films.

Figure 5 shows the plot of the experimental absorption spectra of (a) SnS_2_ and (b) SnS films at room temperature. The spectra show an indirect nature of SnS_2_ and SnS films in this study. The band gap energies (E_g_) for SnS_2_ and SnS films can be determined from the plot of the square root of the absorption coefficient versus the photon energy [48]. The obtained band gap energy values of SnS_2_ and SnS films at room temperature are 2.24 and 1.20 eV, respectively. The values of the determined band gap in this study are in a reasonable agreement with the reported literature [26,49,50,51].

**Figure 6:**
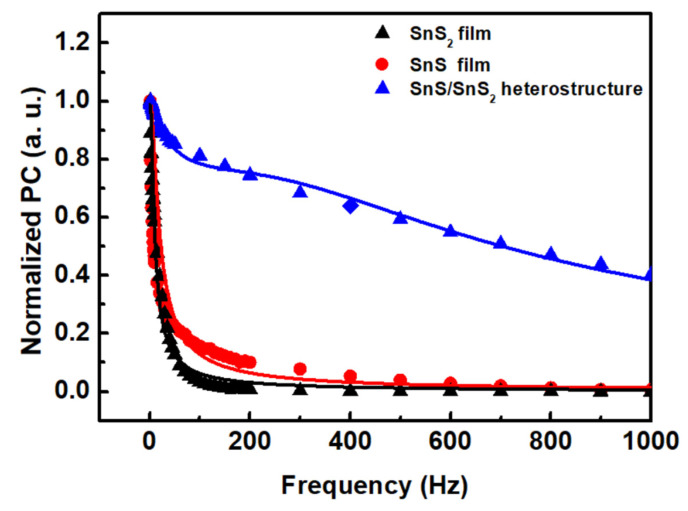
Normalized photoconductivity as a function of frequency of SnS_2_, SnS films, and SnS/SnS_2_ heterostructure.

In order to study the optical response behavior, we performed frequency dependence photoconductivity experiments on SnS_2_, SnS, and their heterostructure in Figure 6. The frequency dependence of photoconductivity can be described by the relation [52,53]:

(1) $$I_{\mathrm{ac}}/I_{\mathrm{dc}}=k_{1}\times \tan h\left(\frac{1}{4{f\tau }_{1}}\right)+k_{2}\times \tan h\left(\frac{1}{4{f\tau }_{2}}\right)$$

where I_ac_ is the ac component of the photocurrent, I_dc_ represents the steady state photocurrent, $k_{1}\;$ and $k_{2}$ are the amplitude coefficients, and $\tau_{1}$ and $\tau_{2}$ are the carrier time constants of two decay processes. The determined parameters are listed in Table 2. The results indicate that the ratio ($k_{1}$) of the long-time constant decay process of SnS_2_ and SnS is 0.31:0.34. However, the ratio ($k_{1}$ = 0.27) of the long-time constant decay process is improved in the SnS/SnS_2_ heterostructure. The normalized photoconductivity decays to almost zero at frequency ~200 Hz for SnS_2_. For SnS, the normalized photoconductivity is around 0.1 at frequency ~200 Hz. This is due to the fact that the carrier time constants of the two decay processes of SnS are faster than SnS_2_. For the SnS/SnS_2_ heterostructure, the determined carrier time constant is faster than for SnS and SnS_2_ films. However, the carrier transport kinetics of the SnS/SnS_2_ heterostructure is significantly enhanced, perhaps due to the improved additional trap state in the interface, which causes a shorter time-constant decay process.

**Figure 7:**
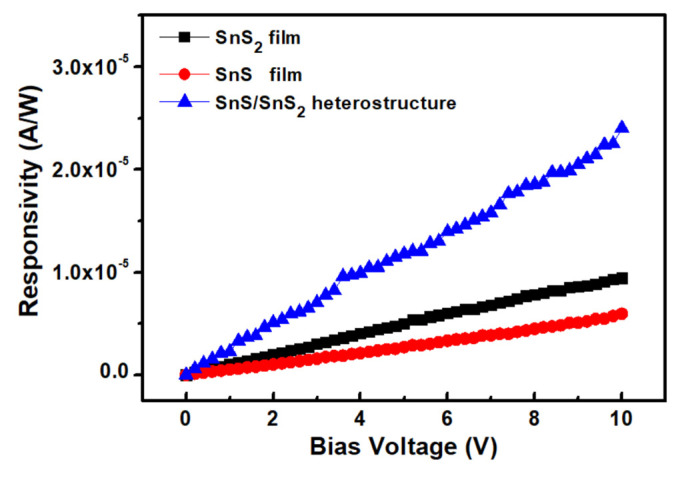
Bias voltage−dependent photoresponsivity of SnS_2_ and SnS films and SnS/SnS_2_ heterostructure.

Further studies of the photoelectrical response properties of (a) SnS_2_ films, (b) SnS films, and (c) the SnS/SnS_2_ heterostructure are shown in Figure 7. The bias voltage-dependent photoresponsivity experiments at the 405 nm excitation light source were measured. As seen in Figure 7, the photoresponsivity increased gradually with the increasing bias voltage. Under a high bias voltage, the electron–hole pairs generated by the excitation light source can be more efficiently separated with an increasing drift velocity, resulting in the high photoresponsivity. The observed linear photoresponsivity depends on the bias voltage, which indicates the ohmic contact between the SnS_2_ and SnS films and the SnS/SnS_2_ heterostructure. The photoresponsivity of SnS/SnS_2_ heterostructure at 10 V is improved several times compared to SnS_2_ and SnS films. This might due to the improved interface properties at the SnS/SnS_2_ heterostructure.

**Figure 8:**
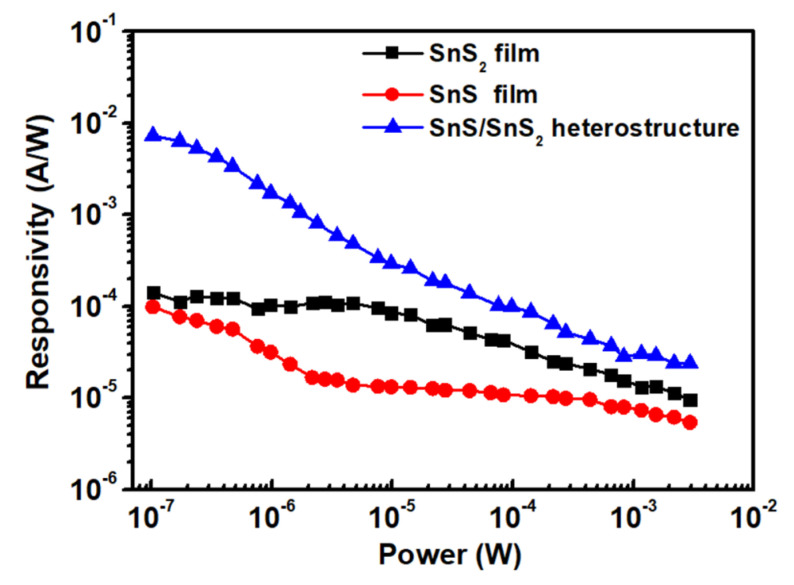
Photoresponsivity of SnS_2_ and SnS films and SnS/SnS_2_ heterostructure as a function of illumination power intensity.

In Figure 8, the power-dependent photoresponsivity of the (a) SnS_2_ and (b) SnS films and the (c) SnS/SnS_2_ heterostructure at 405 nm under a bias voltage of 10 V were performed. The responsivity decayed with the increasing illuminated light intensity. The observation of the reduced responsivity in this study might come from the trap states, such as defects and charged impurities, in the SnS_2_, SnS, and SnS/SnS_2_ heterostructure films. It is known that the built-in electric field could easily enhance the carriers’ transport at the junction. The enhanced photoresponsivity was attributed to the existence of a built-in electric field at the SnS/SnS_2_ heterostructure interface [54]. The stacking SnS/SnS_2_ heterostructure not only improves the photoresponsivity but also benefits the extending of the response range from 1 to 3 eV covering the visible–NIR range. In the condition with low illumination light intensity, the trap states could capture the photo-generated carriers, which avoid the electron-hole pair recombination. As the illumination light intensity becomes higher and higher, most of the electron–hole pair recombination becomes dominated as the number of photo-generated carriers capture by the trap states is limited. Thus, the power-dependent illumination shows the trend of decreased photoresponsivity correlating with the saturation of trap states under a higher light illumination intensity [55,56,57]. Table 3 summarizes the photoresponsivity measured in this work. From the table, we can observe that the photoresponsivity of SnS_2_ and SnS are 0.14 × 10^−3^ A/W and 0.09 × 10^−3^ A/W, respectively, in this work, which is smaller than the reported values of SnS_2_ (0.21 × 10^−3^ A/W) [58] and SnS (4 × 10^−3^ A/W) [59]. This might be due to the larger-scale sample size, which may have more defects, thus lowering the quality of the film. However, the performance could be efficiently improved by the SnS/SnS_2_ heterostructure with the photoresponsivity ~7.31 × 10^−3^ A/W, which might come from the extending broadband response range. The results showed that the photoresponsivity could be enhanced about 7 times by introducing the heterostructure in the present study. It is noticed here that detectivity is also an important characteristic for photodetection devices. However, in this work, our heterostructure is in the size of 10 × 10 mm^2^ and the electrode spacing is 2 mm, which is about 100 to 1000 times that of the standard fabrication (2~20 um). The detectivity will be deteriorated due to the sample size dimension. Our main contribution in this work is to propose a large-size SnS/SnS_2_ heterostructure fabricated by CVD; we discover that its photoresponsivity can be enhanced by introducing the heterostructure. The further investigation of detectivity characteristic improvement with the SnS/SnS_2_ heterostructure could be another valuable study topic.

## 4. Conclusions

In this study, we first demonstrated the growth of a large-area SnS/SnS_2_ heterostructure on glass substrates by the chemical vapor deposition method. The films and heterostructure were characterized by XRD, Raman spectroscopy, and FESEM techniques. Frequency-dependence photoconductivity was used to analyse the carrier transport kinetics in SnS/SnS_2_ heterostructure. The SnS/SnS_2_ heterostructure demonstrated a shorter time-constant decay process ratio of 0.729, accompanied by a time constant of 4.3 × 10^−4^ s. The improvement of the carrier decay process might be due to the improved additional trap state in the interface, which causes a shorter time-constant decay process. The power-dependent photoresponsivity shows that a deteriorated response with increasing illumination intensity can be correlated with the saturation of trap states under a higher light illumination intensity. The results reveal that the photoresponsivity of the SnS/SnS_2_ heterostucture has been enhanced to 7.31 × 10^−3^ A/W, which is about seven times larger than that of individual films. The findings of this study suggest that the SnS/SnS_2_ pn heterostructure exhibits improved photodetection properties, indicating its potential for use in optoelectronic devices. The further improvement of the photoresponsivity performance of SnS/SnS_2_ pn heterostructure could be a further research topic.

## Figures and Tables

**Table 1:** SnS_2_ and SnS films deduced from XRD.

	Lattice Constant (Å)		
	*a*	*b*	*c*
SnS	4.302	11.325	4.003
SnS_2_	3.628	3.628	5.906

**Table 2:** The obtained values of coefficients from the least-square fits to Equation (1) for SnS_2_, SnS, and the SnS/SnS_2_ heterostructure.

	k_1_	k_2_	τ_1_ (s)	τ_2_ (s)
SnS	0.341	0.659	0.043	0.015
SnS_2_	0.316	0.684	0.077	0.032
SnS/SnS_2_	0.271	0.729	0.011	4.3 × 10^−4^

**Table 3:** Comparison of photoresponsivity for SnS_2_, SnS, and SnS/SnS_2_ heterostructure.

Sample	Responsivity (A/W)	References
SnS_2_	0.14 × 10^−3^	this work
SnS_2_	0.21 × 10^−3^	ref [58]
SnS	0.09 × 10^−3^	this work
SnS	4 × 10^−3^	ref [59]
SnS/SnS_2_	7.31 × 10^−3^	this work

## Data Availability

The data presented in this study are available in this article.

## Sample Availability

The samples of the compounds are not available from the authors.
